# Three Column Cervical Fracture-Dislocation in a 3-Year-Old Boy

**DOI:** 10.7759/cureus.23213

**Published:** 2022-03-16

**Authors:** Sananthan Sivakanthan, Abdullah Feroze, Jessica Eaton, Rajiv Saigal

**Affiliations:** 1 Department of Neurological Surgery, University of Washington, Seattle, USA

**Keywords:** posterior cervical surgery, cervical spine fracture, axis fracture: cervical spine trauma, pediatric fractures, pediatric spine

## Abstract

Complete traumatic cervical fracture-dislocation with spinal cord transection in children is a rare entity with no evidence-based guidelines on management. The authors reviewed the literature for pediatric spinal cord injury and present the case of a 3-year-old with traumatic cervical fracture-dislocation and spinal cord transection who presented as a cervical-6 complete spinal cord injury (ASIA A). His other organ systems injured included liver, spleen, bowel, and abdominal aortic injury. The patient underwent halo placement for preoperative reduction followed by open reduction and internal fixation with posterior segmental instrumented fusion. Intraoperatively, the patient had motor evoked potential signals present below the level of his injury. Early postoperative follow-up demonstrated that, although his leg function did not improve, he did demonstrate improvement in upper extremities. This is a rare case of complete cervical spinal cord transection in a pediatric patient. We elected to manage this challenging case with initial external reduction and orthosis with a halo vest followed by acute posterior cervical fusion. Despite a cervical-6 injury level on clinical exam, there was electrographic evidence of function below that level on intraoperative neuromonitoring. Postoperatively the patient has recovered some lost function.

## Introduction

Spinal cord injury in the pediatric population is relatively rare but can be devastating to both the patient and their loved ones. The exact incidence of pediatric spinal cord injury is unknown, however, the National Spinal Cord Injury Statistical Center estimates they represent at least 4% of all spinal cord injuries in the United States. The incidence of spinal cord injury increases rapidly with age with more than 30% of injuries occurring between ages 17 and 23 and more than 50% of injuries occurring between 16 and 30 [1]. Due to this paucity of data, traumatic spinal cord injuries in children can pose a complex clinical problem. From the limited current published literature, it has been shown that there is good potential for recovery of function with both incomplete and complete injuries in the pediatric population, however, there is no direct comparison to the adult population. There are two particularly unique clinically relevant features in pediatric spinal cord injury. The first is the development of post-traumatic scoliosis which is especially common when the spinal cord injury occurs before the patient’s growth spurt [2]. Spinal cord injury without radiographic abnormality (SCIWORA) is the second unique aspect of pediatric spinal cord injury [3].

Of the spectrum of spinal cord injury, complete transection is the rarest due to the shear velocity and magnitude of the forces required to produce such an injury. In fact, survival after complete lower spinal cord injury is uncommon among pediatric patients. Modern advancements in safety have thankfully been effective in reducing the incidence of such injuries. A consequence of being such a rare injury is that when encountered, this can become a challenging problem with no evidence-based recommendations. Thus, in this case report, we present an unfortunate child with a complete cervical transection and our management.

## Case presentation

**Figure 1:**
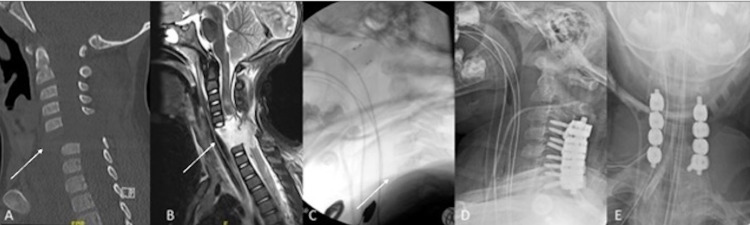
Pre- and postoperative imaging. A: Preoperative CT demonstrating C5/C6 fracture-dislocation (arrow); B: preoperative MRI redemonstrating C5/C6 fracture-dislocation and complete transection of the spinal cord (arrow); C: intraprocedural halo reduction fluoroscopy demonstrating realignment of vertebral bodies (arrow); D: postoperative lateral X-ray; E: postoperative anteroposterior X-ray

The patient is a 3-year-old boy who was involved in a high-speed motor vehicle collision. It is unknown if he was wearing a seat belt or in a child safety seat. He was reportedly ejected from the vehicle. He was found to be “Focused Assessment with Sonography in Trauma” (FAST)-positive on the initial trauma survey and thus taken emergently to the operating room for emergent exploratory laparotomy. CT of the spine revealed a C5-C6 cord transection with traumatic fracture-dislocation (Figures 1A-1B). His other traumatic injuries included a liver laceration grade 4, splenic laceration, bowel and mesenteric injuries, and distal infrarenal abdominal aortic injury with intraluminal thrombus.

After his exploratory laparotomy, his abdomen was kept open. On examination, he was consistent with an American Spinal Cord Injury Association (ASIA) A injury at the C6 level. He was placed in a halo cervical immobilization vest in preparation for OR. During halo placement, under direct live fluoroscopic visualization, we reduced the fracture to near-anatomic alignment (Figure 1C). We then took him to the operating room the following day for a C4-C7 posterior instrumented fusion with intraoperative neuromonitoring of motor evoked potentials (MEPs) and somatosensory evoked potentials (SSEPs). In the OR, the front part of the halo vest was removed. While keeping the head neutral, he was turned prone on a Jackson table with blanket rolls under the chest and torso. An adapter for the halo was attached to the Mayfield head holder. We took a lateral X-ray to check head and neck position. We then loosened the Mayfield head holder and applied compression to further reduce the C5-6 fracture-dislocation. A new X-ray revealed improved alignment (Figures 1D-1E).

After exposure, the site of fracture-dislocation and spinal cord transection at C5-6 was clearly visualized. From C4-C6, we placed bilateral lateral mass screws. Using anatomic landmarks, trajectories were drilled with a pneumatic drill and 1.8 mm matchstick drill bit and then tapped with a custom cervical tap. Given the small anatomy, 3.0 mm diameter screws with 8 or 10 mm length were specially brought in for the case. At C7 on the left, we did not appreciate a good purchase with our initial lateral mass screw and thus we placed a pedicle screw instead. At C7 on the right, we placed a standard lateral mass screw. We covered the site of spinal cord transection with a small piece of collagen dural substitute and then covered this with a dural sealant. We saw no further cerebrospinal fluid (CSF) leak. We decorticated all the bony surfaces at C5-6. We harvested autograft from the spinous processes of C4-C5 and C6. We combined this with 2 mL of demineralized bone matrix and placed this over the decorticated C5-6 posteriorly. 3.5 mm titanium rods were placed over the tulip heads bilaterally. We first tightened the C6 and C7 setscrews bilaterally, then used a persuader to reduce the fracture at C5-6 and tighten the C5 and C6 setscrews bilaterally. An updated X-ray revealed an excellent reduction of the fracture.

**Figure 2:**
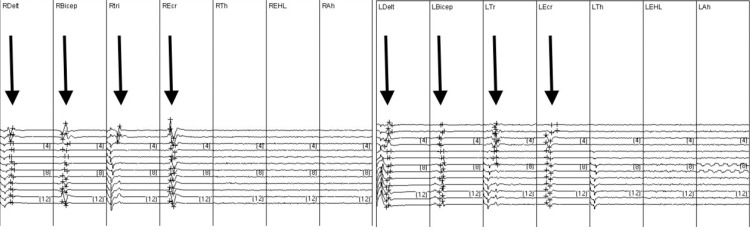
Intraoperative neuromonitoring. Right (R) and left (L) intraoperative electrophysiological recordings shown from deltoid (Delt), biceps (Bicep), triceps (Tri), extensor carpi radialis (Ecr), thenar (Th), extensor hallucis longus (EHL), and abductor hallucis (Ah). Firing can be seen in all muscle groups until the extensor carpi radialis (arrows). The number in brackets refers to the scale in Hz

Intraoperative neuromonitoring demonstrated absent SSEPs in median, ulnar, and tibial nerves as well as in bilateral lower extremities. MEPs were present from the deltoid, biceps, triceps, and extensor carpi radialis only (Figure 2). There were no intraoperative changes in motor signals and the procedure was uneventful. Postoperatively the vest was removed, and the patient was maintained in a cervical collar. The remainder of his postoperative course was uncomplicated. A tracheotomy was performed in addition to a gastrostomy tube, however, the patient was able to be weaned from the ventilator.

At three months postoperatively, the patient is recovering in rehab. Although he has not recovered any leg function, he is able to grip a spoon and lift his arms antigravity.

## Discussion

Cervical spinal cord injuries are among the highest economic and quality of life impact pathologies in all of medicine. They create significant financial and personal burden on patients, caregivers, and the healthcare system. Spinal cord injury can broadly be characterized as complete or incomplete. Complete injuries demonstrate no motor or sensory function below the level of injury whereas incomplete demonstrate some preserved movement or sensation. The American Spinal Injury Association developed a scale to grade SCI severity [4]. Complete spinal cord injury of the cervical spine, such as the case of our patient, represents the most severe type in the spectrum of SCI. This is evident in recovery prognostication as most patients with incomplete SCI show recovery whereas more than 75% of patients with complete spinal cord injury do not improve [5].

The fundamental biomechanical difference between pediatric and adult spinal trauma is that the pediatric spine is intrinsically more elastic than the adult spine, especially in the first 8 years of life. This affects multiple features of the pediatric spine. First, the facet joints are more shallow and oriented horizontally. This increases translational mobility and movement. Second, the spinal ligaments and joint capsules can withstand significant stretch which results in increased subluxation and the occurrence of pseudosubluxation. Third, anterior wedging of the vertebral bodies allows for ventral slippage between motion segments. Finally, the uncinate process is absent and nuchal muscles are generally weaker. These factors in conjunction with a disproportionately large head that adds weight to the fulcrum of the cervical spine change the biomechanical response of the pediatric spine to traumatic insults.

We reviewed three prior reports of complete cervical spinal cord injuries in children (Table 1), two of which had associated distraction injuries. Davern et al. [6] reported a 3-year-old child who was involved in a motor vehicle collision but was restrained in a car seat and suffered a complete spinal cord injury associated with a C6-7 distraction and avulsion of the spinal cord. Their patient underwent halo reduction followed by a single-stage anterior/posterior fixation. At 22 months postop, the patient was neurologically unchanged. Matsumoto et al. [7] reported their case of an 18-month-old girl who had a complete discoligamentous disruption with cervical spinal cord transection at C5-6. Although the details of the extent of recovery are unclear, at 10 years the patient was able to attend elementary school and was mobile with the use of a wheelchair. Finally, Ramrattan et al. [8] reported a case of a 15-month-old girl who was ejected from a car seat during a motor vehicle collision (MVC). The patient suffered C6-7 facet subluxation and interspinous widening and had a complete C6 ASIA A injury. They performed a C5-C7 anterior fusion. At 6 years, the patient was able to move her upper extremities, write her name and even go horseback riding.

**Table 1:** Pediatric spinal cord injury literature review MVC: motor vehicle collision; ASIA: American Spinal Cord Injury Association

Author	Age	Sex	Mechanism	Injury	Clinical Presentation	Surgical Approach	Recovery
Davern et al. [6]	3 years	M	MVC restrained in car seat	C6/7 distraction with complete avulsion of spinal cord	No motor function in any extremity	Halo reduction with neuromonitoring followed by single-stage C6/7 anterior fixation and C5-T2 posterior fusion	Halo maintained for 6 months postoperatively. At 22-month postop patient was neurologically unchanged
Ramrattan et al. [8]	15 months	F	MVC ejected from car seat	C6/7 facet subluxation with interspinous widening	C6 ASIA A	Anterior cervical fusion from C5 to C7	6 years postoperatively the patient is able to “do some horseback riding…move the upper extremities and even able to write her own name”
Matsumoto et al. [7]	18 months	F	MVC ejected from car seat	C5/6 distraction and complete cord transection	C5 ASIA A	C5-C6 posterior fixation	10 years postoperatively patient “attends elementary school…able to speak and operate a wheelchair”

The surgical options, in this case, are varied and complex. In contrast to typical uses of traction, cervical compression was indicated to reduce the fracture. A halo vest was placed at the bedside and used for temporary immobilization until the patient was hemodynamically stable for surgery. Because of the patient’s polytraumatic injuries and instability, emergent spine surgery was not an option. Fluoroscopic visualization confirmed fracture reduction. An additional benefit of external immobilization is the ability to “secure” the patient’s spine so that routine care can occur (such as nursing care, turns, other surgical procedures, etc.). With regards to the selection of a surgical approach, the primary decision point is whether to perform an anterior-only approach, posterior-only approach, or combined approach. In general, this decision should be dictated by the overall stability of the spine and which spinal columns are affected. In children, the growth potential of the spine and understanding if the spine is mature enough to accept screws must be taken into consideration. In the growing child, implanted screws and rods can be explanted after the fracture heals to allow for continued growth. Explant time varies by surgeons, but is often completed 6 months to 1 year after surgery. By age 10, the cervical spine has almost reached the adult height and thus surgery is less likely to lead to malalignment [9]. Furthermore, most of the growth potential also lies in the endplates of the vertebral bodies, and thus care must be taken during an anterior approach to not remove the cartilaginous endplates.

## Conclusions

In this article, the authors report a rare case of cervical fracture-dislocation with spinal cord transection in a 3-year-old child. This is a highly debilitating injury and poses significant challenges in management. In our review of the literature, only three such cases have been previously presented. Prior reports have presented anterior/posterior and anterior-only surgical intervention. This article demonstrates this complex pathology successfully treated by posterior-only fixation. We also demonstrate that the patient had electrographic evidence of neurologic function below the level of the injury and did show some improvement postoperatively.
